# Progressive erythrocytosis under lenvatinib treatment in patients with advanced hepatocellular carcinoma

**DOI:** 10.1007/s00280-023-04519-6

**Published:** 2023-03-24

**Authors:** Laurence Legros, Alina Pascale, Catherine Guettier, Pirayeh Eftekhari, Yasmina Ben Merabet, Maryse Stang, Rachel Bossevot, Emma Goldschmidt, Ayhan Ulusakarya, Stephane Morisset, Maïté Lewin, Didier Samuel, Olivier Rosmorduc

**Affiliations:** 1 Department of Clinical Hematology, Hôpital Bicêtre, AP-HP, 78 Rue du Général Leclerc 94270, Le Kremlin-Bicêtre, France; 2 INSERM UMRS-MD-1197, Université Paris-Saclay, Villejuif, France; 3 France Intergroupe Syndromes Myéloprolifératifs (FIM), Paris, France; 4 Hepato-Biliary Department, Hôpital Paul Brousse, AP-HP, 12-14 Avenue Paul Vaillant Couturier, 94800 Villejuif, France; 5 Anatomic Pathology Department, Hôpital Bicêtre, AP-HP, Le Kremlin-Bicêtre, France; 6 Medical Oncology Department, Hôpital Paul Brousse, AP-HP, Villejuif, France; 7 Independent Biostatistician, Pérouges, France; 8 Radiology Department, Hôpital Paul Brousse, AP-HP, Villejuif, France; 9 INSERM U1193, Université Paris-Saclay, Villejuif, France; 10 Sorbonne Université, Paris, France

**Keywords:** Hepatocellular carcinoma, Lenvatinib, Antiangiogenic drugs, Erythrocytosis, Erythropoietin, Thromboprophylaxis

## Abstract

**Purpose:**

This manuscript reports on the occurrence of early and frequent erythrocytosis in advanced hepatocellular carcinoma (HCC) patients treated with lenvatinib.

**Methods:**

A cohort of 23 patients with advanced HCC, treated with this antiangiogenic drug for at least one month, was retrospectively analyzed.

**Results:**

These patients (82.7% men, median age 58.3, cirrhosis in 60.8%) were treated between October 2019 and September 2020 with lenvatinib, as first-line systemic therapy for 82.6% of them. For 20 patients (87%), an early and significant increase in hemoglobin (Hb) level, up to 1.41 g/dL (*p* < 0.001) was reported and remained elevated. Ten patients (43.5%), all men, reached erythrocytosis (Hb > 16.5 g/dL), 7 were treated with low-dose aspirin for primary thromboprophylaxis and 2 needed phlebotomy. None underwent thromboembolic complications. A significant Hb decrease was observed after treatment discontinuation (*p* < 0.05). Erythropoietin (EPO) serum levels also increased, which was attributed to HCC after immunostaining for EPO in liver biopsies. The Naranjo adverse drug reaction probability scale documented the relationship between erythrocytosis and lenvatinib and regression at treatment discontinuation. Erythrocytosis was hypothesized to be a class effect of anti-VEGF therapies, the magnitude of which might depend on the IC50 value of each molecule.

**Conclusion:**

This report documents the frequent occurrence of erythrocytosis during lenvatinib treatment for advanced HCC, likely secondary to EPO secretion by tumor cells through the antiangiogenic activity levatinib. An early and close monitoring of hematologic parameters is, thus, recommended, together with thromboprophylaxis by low-dose aspirin and phlebotomy in case of symptomatic erythrocytosis.

## Introduction

Liver cancer, hepatocellular carcinoma (HCC) being the most common type in adults, is the third leading cause of cancer-related death and the sixth most commonly diagnosed cancer worldwide with approximately 900,000 new patients each year [1]. About 80–90% of all HCC patients also present with an underlying cirrhosis of different etiologies [2]. Current HCC curative treatments include surgical resection, percutaneous ablation and liver transplantation, resulting in a 5-year survival rate of over 50%. For palliative treatments, a few systemic therapies have been shown to provide survival benefits in patients with advanced HCC in phase III clinical trials (i.e., sorafenib, lenvatinib, atezolizumab plus bevacizumab in first line, regorafenib, cabozantinib in second line) and have, therefore, been approved worldwide [3–7].

Lenvatinib is a tyrosine kinase inhibitor targeting angiogenesis and tumor proliferation through the inhibition of the tyrosine kinase activity of VEGFR 1–3, PDGFR-alpha, fibroblast growth factors receptor (FGFR) 1–4, RET, and mast/stem cell growth factor receptor (KIT) [8, 9]. The side effects of lenvatinib, reported in the pivotal HCC trial are mainly arterial hypertension, proteinuria, fatigue, diarrhea, palmar-plantar erythrodysesthesia syndrome and hypothyroidism.

We recently observed in two patients under lenvatinib treatment for advanced HCC an unexpected increase in hemoglobin (Hb) level, leading to a significant erythrocytosis and a subsequent thrombotic risk. Based on this observation, our cohort of patients with advanced HCC, treated by lenvatinib was retrospectively analyzed. This confirmed a frequent and specific increase in Hb level in this population of patients, justifying close monitoring and prophylactic treatment of the thrombotic risk.

## Methods

### Patients

Data from patients treated with lenvatinib for an advanced HCC between October 2019 and September 2020, in the Hepato-Biliary Center, Paul Brousse Hospital, AP-HP, before the approval of atezolizumab and bevacizumab, were retrospectively examined. The treatment was initiated after discussion in the local multidisciplinary tumor board. Patients aged > 18 years who were treated with lenvatinib for at least 1 month and with at least 3 months follow-up were eligible. Cirrhotic patients had a compensated liver disease (Child–Pugh score A5 or A6). HCC staging was determined according to the Barcelona Clinic Liver Cancer (BCLC) classification. All patients received oral lenvatinib once daily. The treatment was initiated at a dose depending on the patient’s body weight: 8 mg/d below 60 kg and 12 mg/d if 60 kg or more (according to the drug’s summary of product characteristics [SmPC] and based on the pivotal clinical trial). Side effects were recorded according to the Common Criteria Terminology for Adverse Events 4.0. Tumor response was assessed according to RECIST 1.1 and mRECIST criteria, using computed tomography or magnetic resonance imaging at baseline and every 8 weeks under treatment [10]. Treatment was discontinued in case of progression or grade 3 or 4 toxicity. All patients signed informed consent.

### Evaluation of biological parameters

Blood cell counts were performed at treatment initiation, then monthly during treatment and two months after treatment discontinuation. According to the WHO 2016 classification [11], erythrocytosis was considered when the Hb level was over 16 g/dL or the hematocrit (Ht) over 48% in women and, respectively, 16.5 g/dL or 49% in men. Erythropoietin (EPO), ferritin as well as B9 and B12 vitamin levels were retrospectively assessed, when samples were available, obtained before and under lenvatinib treatment. *JAK2* mutation genetic analysis was performed after patient consent to rule out a genetic cause of polycythemia.

### Anti-EPO immunostaining

Four patients had a liver surgical resection or liver transplantation after the initiation of lenvatinib. Among them, two also had pretreatment surgical liver specimens available. Immunohistochemical staining was performed using an anti-EPO monoclonal antibody (Abcam EPO/1367, Cambridge, UK) on deparaffinized sections of tumoral/non-tumoral samples from liver specimens with the LSAB method in a Bond Leica immunostainer (Nanterre, France) at an antibody dilution of 1/400, with protocol ER2 allowing 5 mn for antigenic restoration.

### Statistical analyses

Means, standard deviation, and frequencies were used for data descriptive statistics. Statistical analyses were performed in SPSS 18.0 using Student’s t test, Pearson/Spearman correlations and Wilcoxon Ranks test. The level of significance was set at 5%.

### Pharmacovigilance survey

To assess the probability of adverse drug reactions (ADR), the Naranjo ADR Probability Scale [12] was used. The Naranjo algorithm assesses the causal relationship between a suspected drug and an ADR by answering yes/no/do not know to ten successive questions. The process results in a final score ranging from 4 to 13, allowing the qualification of drug responsibility by four categories: ‘doubtful’ (score 0), ‘possible’ (score between 1 and 4), ‘probable’ (score between 5 and 8), and ‘almost certain’ (score ≥ 9).

The World Health Organization’s pharmacovigilance database of individual-case-safety-reports (ICSR) of adverse drug relation (ADR), VigiBase, was used to identify cases of erythrocytosis and polycythemia complicating lenvatinib therapy and other VEGFR inhibitors [13]. On December 15, 2021, three preferred terms (PT) were queried, respectively, polycythemia, Hb increase, Ht increase as well as substances known for VEGFR inhibition, respectively, lenvatinib, axitinib, bevacizumab, cediranib, pazopanib, regorafenib and semaxanib.

## Results

Twenty-three patients were treated for unresectable HCC with lenvatinib for at least one month in our center between October 2019 and September 2020. Patient characteristics before lenvatinib initiation are summarized in Table 1. Briefly, their median age was 58.3 years (range: 19–79) and 19 patients were males (82.7%). Most had an ECOG performance status score of 0 (78.2%). The majority had cirrhosis (60.8%) and four were treated for recurrent HCC after liver transplantation (17.4%). The two main causes of the underlying liver disease were alcohol consumption (34.7%) and chronic viral infections (HBV/HCV) (34.7%). The HCC Barcelona Clinic Liver Cancer (BCLC) stage was, respectively, C in 56.5% and B in 34.7% of the patients. Two patients presented a fibrolamellar HCC subtype. For conventional HCC, the tumor grade, according to WHO 2019 classification, was well-differentiated (Edmondson grades I–II) in 21.7% of the cases and poorly differentiated (Edmondson grades III–IV) in 26%. Of note, two HCC presented a macrotrabecular-massive subtype. Nineteen patients (82.6%) were treated with lenvatinib as first-line systemic therapy. Among them, six did not receive any previous loco-regional treatment. Four patients (17.4%) received lenvatinib beyond first line.

**Table 1:** Patient characteristics at baseline

Variable	Lenvatinib-treated patients (*n* = 23)
Median age (SD)—years	58.3 (14.28)
Gender—nb. (%)	
Male	19 (82.6)
Female	4 (17.4)
ECOG performance status score—nb. (%)	
0	18 (78.2)
1	5 (21.7)
Cirrhosis—nb. (%)	14 (60.86)
Post-LT—nb. (%)	4 (17.4)
Etiology of the underlying liver disease—nb. (%)	
Alcohol	8 (34.7)
Viral (HBV/HCV)	8 (34.7)
Metabolic	4 (17.4)
Hemochromatosis	2 (8.7)
BCLC (Barcelona Clinic Liver Cancer) stage—nb. (%)	
A	2 (8.7)
B	8 (34.7)
C	13 (56.5)
HCC histological type—nb. (%)	
Edmondson grades I–II	5 (21.7)
Edmondson grades III–IV	6 (26)
Macrotrabecular-massive	2 (8.7)
Fibrolamellar	2 (8.7)
Unknown	8 (34.7)
Prior therapy for HCC—nb. (%)	
Loco-regional therapy	13 (56.5)
Systemic therapy	4 (17.4)
None	6 (26)

*LT* liver transplantation

As shown in Table 2, the median duration of lenvatinib treatment was 181.9 days (SD: 141.6). The initial dose of lenvatinib was 8 mg/d in 65.2% of the patients and 12 mg/d in 34.7%. A modification of lenvatinib dosage was necessary in 56.5% of the cases because of adverse events (AEs). The most frequent AEs were hypertension in 60.8% of the cases and diarrhea in 47.8%.

**Table 2:** Follow-up under Lenvatinib Treatment

Variable	Lenvatinib-treated patients (*n* = 23)
Initial dose of lenvatinib—nb. (%)	
8 mg/d	15 (65.2)
12 mg/d	8 (34.7)
Modification of lenvatinib doses—nb. (%)	13 (56.5)
Median duration of treatment (SD)—days	181.96 (146.23)
Radiological assessment at M2—nb. (%)	
Partial response	5 (22.7)
Stable disease	15 (68.2)
Progression	2 (9.1)
Radiological assessment at M4—nb. (%)	
Partial response	6 (35.3)
Stable disease	8 (47)
Progression	3 (17.6)
Adverse events related to lenvatinib treatment—nb. (%)	
Hypertension	14 (60.8)
Diarrhea	11 (47.8)
PPES	6 (26)
Hypothyroidism	6 (26)
Proteinuria	3 (13)

*M2* two months after starting treatment: one patient stopped treatment before M2, *M4* four months after starting treatment: six patients stopped treatment before M4, *PPES* palmar-plantar erythrodysesthesia syndrome

**Fig. 1:**
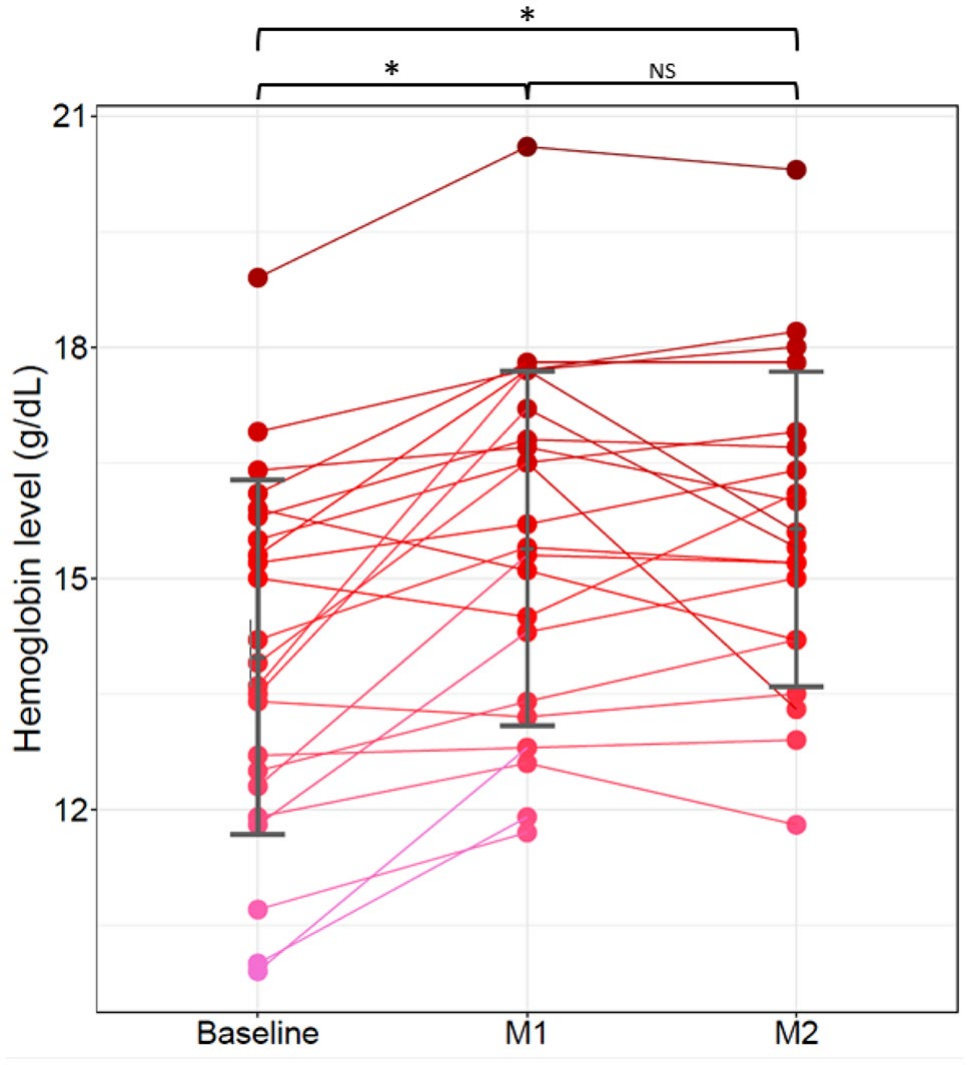
Individual effect of lenvatinib on hemoglobin level per patient over time. Hb levels for each patient at baseline, M1 and M2. Of note, three patients (pink lines and dots) did not have M2 data, one who discontinued lenvatinib after the first month of treatment and missing information for the two others. However, one of those had a blood test at M3 confirming an increased level between M1 and M3 (12.8 g/dL to 14.2 g/dL). Gray bars represent the mean and standard deviation of Hb level at each time point. Between months comparison are represented by square brackets on the top of the figure. (*statistical significance *p* < 0.05; *NS* not significant)

During follow-up, twenty patients (87%) presented with an early and significant increase in Hb level and Ht under lenvatinib. The mean increase in Hb levels between baseline (M0) and one month after starting lenvatinib (M1) was 1.41 g/dL (*p* < 0.001), as shown in Fig. 1. This significant increase was confirmed after 2 months of treatment (M2) (Fig. 1). There was no significant change in white blood cell and platelet counts under lenvatinib treatment. Erythrocytosis criteria were reached in ten patients (43.5%), all males and asymptomatic.

Serum EPO level was assessed in 4 patients before treatment initiation (M0) and in 5 patients at M1 and M2 of treatment. In all 4 patients, baseline EPO level was within the normal range (4.4–16.6 IU/L) at, respectively, 6.8, 10.4, 16 and 6.8 IU/L. By contrast, increased EPO levels were observed at M1, respectively, 124, 22.5, 22.1 IU/L and 102, 21.4, 20.5 IU/L at M2. This elevated EPO level paralleled the Hb increase. As an example, for one patient, a doubled EPO level, from 10.4 IU/L at M0 to 20.5 IU/L at M2 was associated with an increase in Hb from 15.3 g/dL to 18.2 g/dL.

**Fig. 2:**
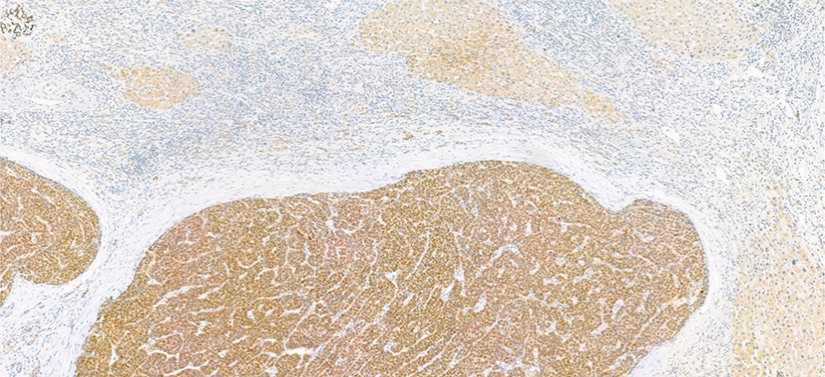
Anti-erythropoietin immunohistochemistry of native liver after lenvatinib treatment demonstrating strong staining of tumor cells compared to a faint positivity in cirrhotic nodules (Immunoperoxidase, magnification X 90)

Immunostaining for EPO in post-treatment surgical specimens demonstrated a strong staining in tumor cells compared to a faint positivity in cirrhotic nodules (Fig. 2), suggesting that the tumor was the source of increased EPO production.

Among the ten patients who developed erythrocytosis, seven (70%) were treated with low-dose acetylsalicylic acid (ASA) for primary thromboprophylaxis. Two required a phlebotomy, as their Ht became higher than 52% with a subsequent high risk of thrombosis. After a median follow-up of 9 months (range: 5–15) under ASA treatment, no patient developed a thromboembolic complication nor a hemorrhagic event.

Regarding treatment efficacy, radiological assessment at M2 showed partial response in 5 patients (22.7%), stable disease in 15 (68.2%) and progression in 2 (9.1%) (Table 2). One patient was not evaluable for response at M2, as treatment was halted due to severe alteration of the performance status. Radiological assessment at M4 of the 17 patients still under treatment showed a partial response in 6 (35.3%), stable disease in 8 (47%) and progression in 3 (17.6%).

At last follow-up, 3 patients had died and 20 were alive, of whom 10 still on lenvatinib treatment. Thirteen had discontinued lenvatinib, 7 for tumor progression, 2 for AEs, one for hyperthyroidism, one for impaired performance status, one for liver failure, and one because of liver transplantation. Twelve patients were evaluable for Hb level and Ht after treatment discontinuation. In all but one, the mean hemoglobin level had decreased of 1.12 g/dL within the first month after treatment discontinuation (*p* < 0.05) (range: 0.2–1.6).

In univariate analysis, there was no correlation between Hb changes and the following criteria: gender, HCC histological subtype, cirrhosis, previous loco-regional treatments, initial dose of lenvatinib (Table 3). No correlation either was observed with adverse events (hypertension *p* = 0.360; proteinuria *p* = 0.963; diarrhea *p* = 0.139; palmar-plantar erythrodysesthesia syndrome *p* = 0.099; hypothyroidism *p* = 0.326), treatment duration (*p* = 0.72) nor response at M2 (*p* = 0.974) and M4 (*p* = 0.181).

**Table 3:** Univariate Cox regression model analysis

Variable	Reference level	Class level	Difference estimation [95% CI]	*p*-value
Age	Continuous variable		− 0.04 [− 0.10–0.03]	0.268
Gender	F	M	2.44 [0.23–4.65]	0.042
HCC histological subtype	Well differentiated	poorly	0.48 [− 1.15–2.10]	0.575
Cirrhosis	Continuous variable		1.34 [− 0.47–3.15]	0.161
Hemoglobin	Continuous variable		0.77 [0.53–1.02]	< 0.001
Previous loco-regional treatments	Y	N	0.00 [− 1.32–1.32]	1.000
Initial dose of lenvatinib	12 mg	8 mg	− 0.11 [− 1.37–1.14]	0.860

For evaluable patients, the Naranjo adverse drug reaction probability scale confirmed an association between erythrocytosis and lenvatinib at probable level. Assessment through the WHO pharmacovigilance database on December 15, 2021 identified 8 ICRS of polycythemia, 8 Hb increases and 2 Ht increases under lenvatinib from a total of 9641 reports. The effects of other VEGFR inhibitors are summarized in Table 4.

**Table 4:** Number of individual case reports according to VEGFR inhibitors in the WHO database

VEGFR inhibitors	Total ADR	Polycythemia	Hemoglobin increase	Hematocrit increase	Total AE
Lenvatinib	9641	8	8	2	18
Pazopanib	25,765	18	16	8	42
Axitinib	10,708	21	16	7	44
Bevacizumab	73,693	18	23	17	58
Sorafenib	29,816	9	5	0	14
Sunitinib	36,552	7	9	4	20
Vandetanib	1290	6	4	4	14
Cediranib	157	0	2	0	2
Regorafenib	13,427	1	2	0	3
Semaxanib	19	0	0	0	0

*ADR* adverse drug reaction; *AE* adverse event

## Discussion

Lenvatinib is an inhibitor of multiple tyrosine kinases including VEGFRs and FGFRs. It was approved by the U.S. Food and Drug Administration (FDA) for the treatment of solid tumors including thyroid cancer (2015), renal cell carcinoma (2016) and more recently in first line for advanced HCC (2018) [4] and in combination with pembrolizumab for endometrial carcinoma (2019). After over 6 years of clinical experience, lenvatinib is described as a well-tolerated drug that causes AEs characteristic of angiogenesis inhibitors, including hypertension (68%), diarrhea (59%), palmar-plantar erythrodysesthesia syndrome (32%), and proteinuria (31%).

Here, we report what is to our knowledge the first series demonstrating the occurrence of early and very frequent erythrocytosis in HCC patients under lenvatinib, with a potential risk of thromboembolic complications. The relationship between erythrocytosis and lenvatinib and its regression at treatment discontinuation, documented by Naranjo adverse drug reaction probability scale, confirmed the direct implication of the drug in these AEs.

Several case reports of erythrocytosis occurring during antiangiogenic treatments in cancer (cediranib, vandetanib, axitinib, pazopanib, sunitinib, sorafenib or bevacizumab) have been published [14–22]. However, the high frequency of this AE (87%) is striking in the present series, despite use of the classical dosage. Interestingly, in comparison with sorafenib, sunitinib or regorafenib, lenvatinib inhibition of VEGFR on an HCC cell line (HepG2), assessed by the IC50 value, was reported to be 4–128 times more efficient [23]. This high VEGFR inhibition could explain the higher frequency of Hb level increase under lenvatinib. It cannot be excluded, however, that erythrocytosis could be a class effect of all anti-VEGF therapies (Table 3), but its magnitude and its frequency might depend on the IC50 value of the different molecules.

Interestingly, this AE of lenvatinib has not been reported in other cancers. For example, in radioactive iodine (RAI)-refractory thyroid cancer which was the first indication of lenvatinib, phase II and III clinical trials did not report any Hb increase [24–26]. Previous RAI treatment, known to induce bone marrow impairment [27–29], could be an explanation, although our results clearly demonstrate the role of HCC tumoral cells in yielding an EPO increase.

Indeed, a liver specificity should be considered. In adults, EPO is produced not only by renal peritubular cells, but also by the liver [30]. In fact, HCC could be associated with a paraneoplastic syndrome characterized by secondary erythrocytosis and high plasma EPO levels produced by the cancer cells [31–33]. Here, immunohistochemistry confirmed that HCC tumoral cells were able to produce higher levels of EPO, under lenvatinib, than non-tumoral hepatocytes in the cirrhotic liver (Fig. 2). Moreover, there is now increasing evidence showing the impact of hypoxia on hepatic EPO production. An increase of EPO secretion by hepatocytes and stellate cells has been reported in rats in hypoxia conditions [34–37]. It has also been shown that VEGF inhibition results in a large increase in EPO secretion from hepatic cells, leading to enhanced erythropoiesis and elevated circulating red blood cell counts [38]. Therefore, lenvatinib, one of the most potent VEGFR inhibitors, could potentiate EPO production by HCC cells subject to hypoxic-like conditions and explain the Hb/Ht increase. The doubling of EPO level under lenvatinib in one patient, leading to a high level of Hb, supports this hypothesis. EPO level monitoring might represent a simple surrogate marker for stringent blockade of VEGFR in HCC patients treated by lenvatinib.

Several reports have suggested that EPO and EPO stimulating agents could promote tumor cell proliferation through its specific receptor (EPOR) and hypoxia in head and neck tumors [39], breast cancer [40] and HCC [41]. This was shown to be associated with poorer overall survival rates in HCC patients [42]. However, other studies contradicted these findings [43, 44] and even suggested that the level of EPOR in the cirrhotic tissue could be correlated with tumor cell differentiation and a favorable outcome [45]. In the present study, erythrocytosis was not correlated with the tumor response nor with the occurrence of other AEs.

Erythrocytosis is associated with a risk of thrombosis in relation with an increase of blood viscosity and blood flow decrease [46–48]. Antiangiogenic drug therapy is also associated with an increased risk of thrombosis [49]. In thyroid cancer patients treated with lenvatinib, arterial thromboembolic events occurred in 5.4% of the cases (2.7% grade ≥ 3) and venous thromboembolic events in 5.4% (3.8% grade ≥ 3) [26]. Furthermore, HCC occurs in 80–90% of the cases in cirrhotic patients, who, due to complex coagulation disorders, present with a pro-thrombotic condition. For all these reasons, a close monitoring of hematologic parameters is recommended before and during lenvatinib treatment. In case of erythrocytosis, we propose to initiate thromboprophylaxis (i.e., low-dose ASA). Control of cardiovascular risk factors such as hypertension, hyperlipidemia, diabetes and smoking cessation should be emphasized if applicable.

In conclusion, in this cohort of HCC patients, a frequent and specific erythrocytosis was evidenced, possibly secondary to EPO secretion by tumor cells related to the antiangiogenic activity of lenvatinib. These results suggest that a close and early monitoring of hematologic parameters should be performed for such patients. Thromboprophylaxis by ASA should be prescribed in case of erythrocytosis and phlebotomy in case of symptomatic effects of erythrosis.

## Data Availability

The data that support the findings of this study are available on request from the corresponding author [LL] upon reasonable request. The data are not publicly available due to privacy restrictions.
